# A Botybirnavirus Isolated from *Alternaria tenuissima* Confers Hypervirulence and Decreased Sensitivity of Its Host Fungus to Difenoconazole

**DOI:** 10.3390/v14102093

**Published:** 2022-09-21

**Authors:** Zhijian Liang, Huihui Hua, Chunyan Wu, Tao Zhou, Xuehong Wu

**Affiliations:** College of Plant Protection, China Agricultural University, Haidian District, Beijing 100193, China

**Keywords:** *Alternaria tenuissima*, *Botybirnavirus*, Alternaria alternata botybirnavirus 1 (AaBRV1), hypervirulence, transcriptome, reduced sensitivity to difenoconazole

## Abstract

Alternaria alternata botybirnavirus 1 (AaBRV1) was isolated from a strain of *Alternaria alternata*, causing watermelon leaf blight in our previous research. The effect of AaBRV1 on the phenotype of its host fungus, however, was not determined. In the present study, a novel strain of AaBRV1 was identified in *A. tenuissima* strain TJ-NH-51S-4, the causal agent of cotton Alternaria leaf spot, and designated as AaBRV1-AT1. A mycovirus AaBRV1-AT1-free strain TJ-NH-51S-4-VF was obtained by protoplast regeneration, which eliminated AaBRV1-AT1 from the mycovirus AaBRV1-AT1-infected strain TJ-NH-51S-4. Colony growth rate, spore production, and virulence of strain TJ-NH-51S-4 were greater than they were in TJ-NH-51S-4-VF, while the sensitivity of strain TJ-NH-51S-4 to difenoconazole, as measured by the EC_50_, was lower. AaBRV1-AT1 was capable of vertical transmission via asexual spores and horizontal transmission from strain TJ-NH-51S-4 to strain XJ-BZ-5-1_hyg_ (another strain of *A. tenuissima*) through hyphal contact in pairing cultures. A total of 613 differentially expressed genes (DEGs) were identified in a comparative transcriptome analysis between TJ-NH-51S-4 and TJ-NH-51S-4-VF. Relative to strain TJ-NH-51S-4-VF, the number of up-regulated and down-regulated DEGs in strain TJ-NH-51S-4 was 286 and 327, respectively. Notably, the expression level of one DEG-encoding cytochrome P450 sterol 14α-demethylase and four DEGs encoding siderophore iron transporters were significantly up-regulated. To our knowledge, this is the first documentation of hypervirulence and reduced sensitivity to difenoconazole induced by AaBRV1-AT1 infection in *A. tenuissima*.

## 1. Introduction

Mycoviruses are ubiquitously present in all major groups of fungi [1,2] and are currently classified in 22 taxa by the International Committee on Taxonomy of Viruses (ICTV, https://talk.ictvonline.org/, accessed on 18th September 2021). Members of eight families (*Amalgaviridae*, *Chrysoviridae*, *Megabirnaviridae*, *Partitiviridae*, *Polymycoviridae*, *Reoviridae*, *Totiviridae*, and *Quadriviridae*), as well as one recognized genus *Botybirnavirus*, have double-stranded RNA (dsRNA) genomes. The genus *Botybirnavirus* contains eight members possessing two-fragmented dsRNA genomes, namely Botrytis porri botybirnavirus 1 (BpRV1), Sclerotinia sclerotiorum botybirnavirus 1 (SsBRV1), Sclerotinia sclerotiorum botybirnavirus 2 (SsBRV2), Bipolaris maydis botybirnavirus 1 (BmBRV1), Soybean leaf-associated botybirnavirus 1 (SlaBRV1), Bipolaris maydis botybirnavirus 1 strain BdEW220 (BmBRV1-BdEW220), Alternaria botybirnavirus 1 (ABRV1), and Alternaria alternata botybirnavirus 1 (AaBRV1) [3,4,5,6,7,8,9,10].

Most mycoviruses are associated with cryptic or latent infections of fungal hosts [11,12], however, some mycoviruses are reported to confer hypovirulence or hypervirulence to their host fungi. Viruses that confer hypovirulence are considered as potential biological agents for controlling fungal diseases. For example, Cryphonectria parasitica hypovirus 1 (CHV1) has been successfully used to control chestnut blight disease caused by *Cryphonectria parasitica* [13], and Sclerotinia sclerotiorum hypovirulence-associated DNA virus 1 (SsHADV-1) can infect *Sclerotinia sclerotiorum* colonizing *Brassica napus* and convert it from a pathogenic fungus to a non-pathogenic endophyte that promotes plant growth [14,15]. Mycoviruses conferring hypervirulence have also been identified in multiple pathogenic fungi [16,17,18,19,20]. Examples include a 6.4-kb dsRNA identified in *Rhizoctonia solani* that increases the virulence of its host fungus on potato [16], Aspergillus fumigatus polymycovirus-1 (AfuPmV-1) and a polymycovirus found in *Aspergillus fumigatus* that enhances the virulence of their host fungus [17,18], and Talaromyces marneffei partitivirus-1 (TmPV1) that dramatically increases the virulence, abiotic stress tolerance, and toxicity of its host fungus, *Talaromyces marneffe* [20].

Fungi in the genus *Alternaria* are significant plant pathogens that cause black spot disease on a variety of crops worldwide [21,22]. Sterol demethylation inhibitors (DMIs) fungicides, such as difenoconazole, have been widely utilized to control black spot disease caused by *Alternaria* [23,24]. Strains with lower sensitivity to DMI, however, are prevalent. Several biochemical mechanisms of DMI-resistance have been reported, including mutations in cytochrome P450 sterol 14-alpha demethylase (CYP51) [25], overexpression of CYP51 [26] in field isolates of *Mycosphaerella graminicola*, and pump-out enhancement by ATP-binding cassette transporters (ABC) in *Aspergillus fumigatus* [27] or major facilitator systems (MFS) [28] in *Zymoseptoria tritici*.

Co-infection of Penicillium digitatum polymycovirus 1 (PdPmV1) and Penicillium digitatum Narna-like virus 1 (PdNLV1) [29], or a single infection of Penicillium crustosum chrysovirus 1 (PcCV1) [30] all enhance the sensitivity of their host fungi to prochloraz, supporting the hypothesis that increased sensitivity to DMIs is due to mycovirus infection. Notably, Alternaria alternata chrysovirus 1-AT1 (AaCV1-AT1) reduces the sensitivity of its host fungus *A. tenuissima* to difenoconazole and tebuconazole [31].

Comparative transcriptome analysis of mycovirus-infected and mycovirus-free fungi can aid in the identification of potential functional genes or pathways involved in mycovirus-fungus interactions [32]. Only a few studies, however, have demonstrated transcriptional or translational alterations in mycovirus-infected fungi, including reports on *C. parasitica*, *A. fumigatus*, *S. sclerotiorum*, *Heterobasidion parviporum*, and *Fusarium graminearum* [33,34,35,36,37]. Results from these studies have indicated that mycovirus infection affects a variety of vital biological processes, including primary and secondary metabolism, transcriptional regulation, signal transduction, substance transport, virulence factor expression, and ribosome function.

Alternaria alternata botybirnavirus 1 (AaBRV1) was isolated from *Alternaria alternata*, the causal agent of watermelon leaf blight, in our previous research [10]. The effect of AaBRV1 on the metabolic, physiological, and other biological properties of its host fungus was not determined. In the present study, a novel strain of AaBRV1 was identified from *A. tenuissima* strain TJ-NH-51S-4 causing Alternaria leaf spot in cotton and was named AaBRV1-AT1. The effect of AaBRV1-AT1 infection on colony growth rate, spore production, virulence, and sensitivity to difenoconazole of its host fungus *A. tenuissima* strain TJ-NH-51S-4 were also evaluated. Assays on the vertical and horizontal transmission of AaBRV1-AT1 via asexual spores and through hyphal contact using pairing cultures, respectively, were also conducted. Additionally, a comparative analysis of RNA sequence (RNA-seq) data was utilized to identify differentially expressed genes (DEGs) between the mycovirus AaBRV1-AT1-infected strain TJ-NH-51S-4 and the mycovirus AaBRV1-AT1-free strain TJ-NH-51S-4-VF. The comparative analysis provided information that was used to identify potential molecular mechanisms responsible for causing hypervirulence and a reduced sensitivity to difenoconazole.

## 2. Materials and Methods

### 2.1. Fungal Strains

Five strains of *Alternaria tenuissima* were used in this study (TJ-NH-51S-4, XJ-BZ-5-1, TJ-NH-51S-4-VF, XJ-BZ-5-1_hyg_, and XJ-BZ-5-1_hyg_-V). Strains TJ-NH-51S-4 and XJ-BZ-5-1 were isolated from cotton leaves collected from Tianjin municipality and Xinjiang Uygur autonomous region of China, respectively, that exhibited symptoms of Alternaria leaf spot. The strains were identified to be *A. tenuissima* based on morphological traits and sequence analysis of the internal transcribed spacer region of ribosomal DNA (rDNA-ITS) and histone 3 genes using previously described methods [22]. Sequences of the rDNA-ITS and histone 3 genes of the two strains were deposited in GenBank under the accession numbers OM276061 and OM275828 (for rDNA-ITS), and OM220371 and OM220138 (for histone 3 gene), respectively. The mycovirus AaBRV1-AT1-free strain TJ-NH-51S-4-VF was obtained utilizing protoplast methodology to eliminate the mycovirus AaBRV1-AT1 from the mycovirus AaBRV1-AT1-infected strain TJ-NH-51S-4. A hygromycin B phosphotransferase gene conferring hygromycin-resistance was transformed into strain XJ-BZ-5-1 to obtain strain XJ-BZ-5-1_hyg_. Strain XJ-BZ-5-1_hyg_-V containing the mycovirus AaBRV1-AT1 was obtained through hyphal contact by pairing cultures of colonies of *A. tenuissima* strain TJ-NH-51S-4 (donor strain) and *A. tenuissima* strain XJ-BZ-5-1_hyg_ (recipient strain) on the same potato dextrose agar (PDA) plate. The three strains TJ-NH-51S-4-VF, XJ-BZ-5-1_hyg_, and XJ-BZ-5-1_hyg_-V were verified to be *A. tenuissima* using the methods described above. All five strains TJ-NH-51S-4, XJ-BZ-5-1, TJ-NH-51S-4-VF, XJ-BZ-5-1_hyg_, and XJ-BZ-5-1_hyg_-V were grown on PDA plates at 25 °C in the dark for 7 d for subsequent use.

### 2.2. Extraction and Purification of RNA

Extraction of dsRNA employed the use of CF-11 cellulose (Sigma-Aldrich, China) chromatography as previously described [38]. Extracted dsRNA was treated with DNase I and S1 Nuclease (TaKaRa, Dalian, China) and then evaluated by electrophoresis in 1.0% (*w*/*v*) agarose gel at 120 V and subsequently purified using a gel extraction kit according to the manufacturer’s instructions (Aidlab Biotechnologies, Beijing, China). Total RNA was extracted using TRIzol Reagent (Invitrogen, CA, USA) according to the manufacturer’s instructions. Both dsRNA and total RNA were stored at −80 °C for further use.

### 2.3. Synthesis and Molecular Cloning of Complementary DNA (cDNA)

Purified dsRNA was coupled with a tagged random primer, RACE3RT, for synthesizing the first strand of cDNA using moloney murine leukemia virus (M-MLV) reverse-transcriptase (TaKaRa, Dalian, China) [10]. *Taq* DNA polymerase and dNTPs were used to synthesize the second strand of cDNA, after which the generated cDNA was purified (TaKaRa, Dalian, China). Double-stranded cDNA was ligated into the pTOPO-T vector and transformed into *Escherichia coli* Top10 cells (Aidlab Biotechnologies, Beijing, China). All positive clones with inserts > 500 bp in length were sequenced, and the obtained sequences were used to design specific primers (Table 1) for determining the sequence gaps between clones of cDNA by reverse transcription-polymerase chain reaction (RT-PCR). Terminal sequences of each dsRNA were obtained using the ligase-mediated rapid amplification of cDNA ends (RLM-RACE) technique [4]. Sequencing was performed by the Beijing Tianyihuiyuan Biotechnology Co., Ltd. (Beijing, China), and the whole genome sequence was assembled using DNAMAN 7.0 (Lynnon Biosoft, Montreal, QC, Canada).

### 2.4. Analysis of Sequences and Phylogenetic Tree Construction

ORF Finder (https://www.ncbi.nlm.nih.gov/orffinder, accessed on 18 September 2021) was used to evaluate the open reading frames (ORFs) of the obtained sequences. A conserved domain database (CDD, http://www.ncbi.nlm.nih.gov/Structure/cdd/wrpsb.cgi, accessed on 18 September 2021) was queried to identify conserved motifs. CLUSTAL_X was used to conduct multiple alignments [39]. A phylogenetic tree based on the deduced amino acid (aa) sequence of RNA-dependent RNA polymerase (RdRp) was constructed using the maximum-likelihood (ML) technique in MEGA version 6.0 software with a bootstrap value of 1000 [40]. The reference sequences of viruses used to construct the phylogenetic tree were obtained from NCBI (http://www.ncbi.nlm.nih.gov/genomes, accessed on 18 September 2021).

### 2.5. Elimination of AaBRV1-AT1 from Strain TJ-NH-51S-4

A protoplast regeneration protocol described by Kamaruzzaman et al. [41] was used to eliminate the mycovirus AaBRV1-AT1 from strain TJ-NH-51S-4. Mycelial plugs were randomly cut from regenerated colonies of strain TJ-NH-51S-4 and transplanted to fresh PDA plates for observation of colony morphology. Gel electrophoretic profiles of dsRNA and RT-PCR detection of AaBRV1-AT1 using mycovirus-specific primers (Table 1) were conducted in accordance with previously described methods [41] to determine if the mycovirus AaBRV1-AT1 had been successfully eliminated from strain TJ-NH-51S-4.

### 2.6. Vertical and Horizontal Transmission Assays

Vertical viral transmission via asexual spores was assessed using previously described methods [42]. Briefly, strain TJ-NH-51S-4 was cultured at 25 °C in the dark for 7 d on PDA plates and used to collect asexual spores in sterilized double-distilled water, which were then dispersed on PDA plates at appropriate dilutions. Twenty-four single-spore randomly selected colonies were then individually transferred to new, separate PDA plates and cultured for 7 d at 25 °C in the dark and subsequently used to extract dsRNA. A positive presence of dsRNA in the single-spore cultures derived from strain TJ-NH-51S-4 was used to determine if AaBRV1-AT1 was vertically transmitted via asexual spores.

Horizontal transmission of viral dsRNA segments through hyphal anastomosis was also evaluated using previously described methods [41,42,43,44]. Mycelial agar plugs from the donor strain TJ-NH-51S-4 and recipient strain XJ-BZ-5-1_hyg_ were pairing-cultured for 5 d at 25 °C in the dark on the same PDA plate in close proximity (approximately 10 mm) to each other. The pairing was replicated across three plates. Mycelial blocks from the fungal paired PDA plates were transferred to new PDA plates amended with 50 µg/mL of hygromycin and grown for 7 d at 25 °C in the dark. Mycelial transfers that could grow on PDA plates containing hygromycin were transferred to new PDA plates to purify the derivative strains, which were designated as XJ-BZ-5-1_hyg_-V. Gel electrophoretic profiles of dsRNA and RT-PCR detection of AaBRV1-AT1 using mycovirus-specific primers (Table 1) were conducted as the methods mentioned above in Section 2.5 to confirm if AaBRV1-AT1 had been successfully transmitted from TJ-NH-51S-4 to XJ-BZ-5-1_hyg_.

### 2.7. Effect of AaBRV1-AT1 on the Phenotype of Its Host Fungus

Colony morphology and colony growth rate of strains TJ-NH-51S-4, TJ-NH-51S-4-VF, XJ-BZ-5-1_hyg_, and XJ-BZ-5-1_hyg_-V were evaluated using previously described procedures [31]. The number of conidiospores produced by each colony cultured on PDA plates was assessed under a Nikon Eclipse Ci microscope equipped with a Canon EOS 700D camera with the aid of a hemocytometer according to previously described methods [45].

Pathogenicity assessment of the four strains TJ-NH-51S-4, TJ-NH-51S-4-VF, XJ-BZ-5-1_hyg_, and XJ-BZ-5-1_hyg_-V was conducted on detached, fully expanded healthy cotton (cv. Lumianyan22) leaves using a slightly modified version of the methods reported by Pryor and Michailides [46] and Ma et al. [22]. The four strains were grown on PDA plates at 25 °C in the dark for 7 d, after which agar plugs (5 mm in diameter) were cut from the edge of a colony and placed directly on the upper surface of cotton leaves. The cotton leaves were then placed in a growth chamber at 25 °C, 90% relative humidity (RH) and a 12 h photoperiod per day. The diameter of lesions on cotton leaves was measured at 7 d post inoculation and these values were utilized to calculate the disease incidence and disease index for the four strains.

The sensitivity of the four strains TJ-NH-51S-4, TJ-NH-51S-4-VF, XJ-BZ-5-1_hyg_, and XJ-BZ-5-1_hyg_-V to difenoconazole was evaluated in vitro as described in a previous study [24] with minor modifications. The PDA media were amended with difenoconazole to establish final concentrations of 5.00, 1.00, 0.50, 0.10, and 0.05 µg/mL. The median effective concentration (EC_50_) of difenoconazole for the four strains was calculated using previously described methods [31]. Three replicates were used for each strain-difenoconazole combination, and the experiment was repeated twice. Paired *t*-test was performed using Graphpad Prism version 8.0 for the statistical analysis (*, *p* < 0.05; **, *p* < 0.01; ***, *p* < 0.001; ****, *p* < 0.0001).

### 2.8. cDNA Library Preparation and Transcriptomic Analyses

RNA-seq analysis was performed using total RNA extracted from strains TJ-NH-51S-4 and TJ-NH-51S-VF, which had been cultured on PDA plates for 7 d at 25 °C in the dark. Transcriptome analysis was conducted to determine the effect of AaBRV1-AT1 on gene expression in *A. tenuissima* strain TJ-NH-51S-4.

A cDNA library was constructed of the two strains using the high throughput Illumina strand-specific RNA sequencing library protocol [47]. Total RNA was digested with DNase I to purify total RNA, and then the samples were enriched in mRNA using magnetic beads Oligo (dT). The obtained mRNA was cut into small fragments and first strand cDNA was synthesized using random hexamer primers. Second strand cDNA was synthesized using DNA polymerase I and RNase H. Sequencing adapters were ligated to the short fragments after purification with a QiaQuick PCR extraction kit so that different samples could be distinguished after the sequencing of pooled samples. The final pooled cDNA library was sequenced on an Illumina HiSeq™ 2500 platform at Beijing BioMarker Technologies Co., Ltd. (Beijing, China). Libraries were constructed from three biological replicates of each strain. After sequencing was completed, adaptor sequences, empty reads, and low-quality sequences were removed from the raw reads to generate clean reads.

TopHat [48] software was used to align the high-quality clean read sequences to the reference genome (*A. tenuissima*: txid119927 (Organism: noexp)). Reads mapped to the genome were utilized to determine the expression of each gene in each sample. Annotation of unigenes was performed using the databases described in our previous study [49]. Differentially expressed genes (DEGs) were identified using DESeq [50] software, which utilized statistical methods based on a negative binomial distribution model. DEGs were designated as genes whose expression had a |log_2_fold-change| > 1 and a false discovery rate (FDR), as well as an adjusted *p*-value < 0.05 [49].

### 2.9. Validation of RNA-seq Data Using Reverse Transcription-Quantitative PCR

Reverse transcription-quantitative PCR (RT-qPCR) using gene-specific primers (Table S1) based on the RNA-seq data was designed using Primer Premier Version 5.0 (PREMIER Biosoft International, Palo Alto, CA, USA) software [51]. RT-qPCR was used to validate the expression of eleven selected DEGs related to cytochrome P450, drug resistance, ABC, and MFS (Table S1). RT-qPCR was conducted following the procedure published by Dossa et al. [52], using total RNA extracted from strains TJ-NH-51S-4 and TJ-NH-51S-VF as templates. First strand cDNA was synthesized using HiScript III All-in-one RT SuperMix (Vazyme). RT-qPCR was conducted using a Thermo QuantStudio1 thermocycler with ChamQ Universal SYBR qPCR Master Mix (Vazyme) according to the manufacturer’s instructions. The histone 3 gene (*HIS3*) was used as an internal control. Each reaction was carried out using a 20 µL mixture consisting of 10 µL of 2 × ChamQ Universal SYBR qPCR Master Mix, 6 µL of nuclease-free water, 1 µL of each primer (10 mM), and 2 µL of 20 ng diluted cDNA. The RT-qPCR analysis utilized three biological replicates and was conducted three times. The cycling profile was 95 °C for 30 s, followed by 40 cycles of 95 °C/10 s and 60 °C/30 s. Data are presented as relative transcript levels that were derived using the 2^−ΔΔct^ technique [53].

## 3. Results

### 3.1. Characterization of AaBRV1 in A. tenuissima Strain TJ-NH-51S-4

**Figure 1:**
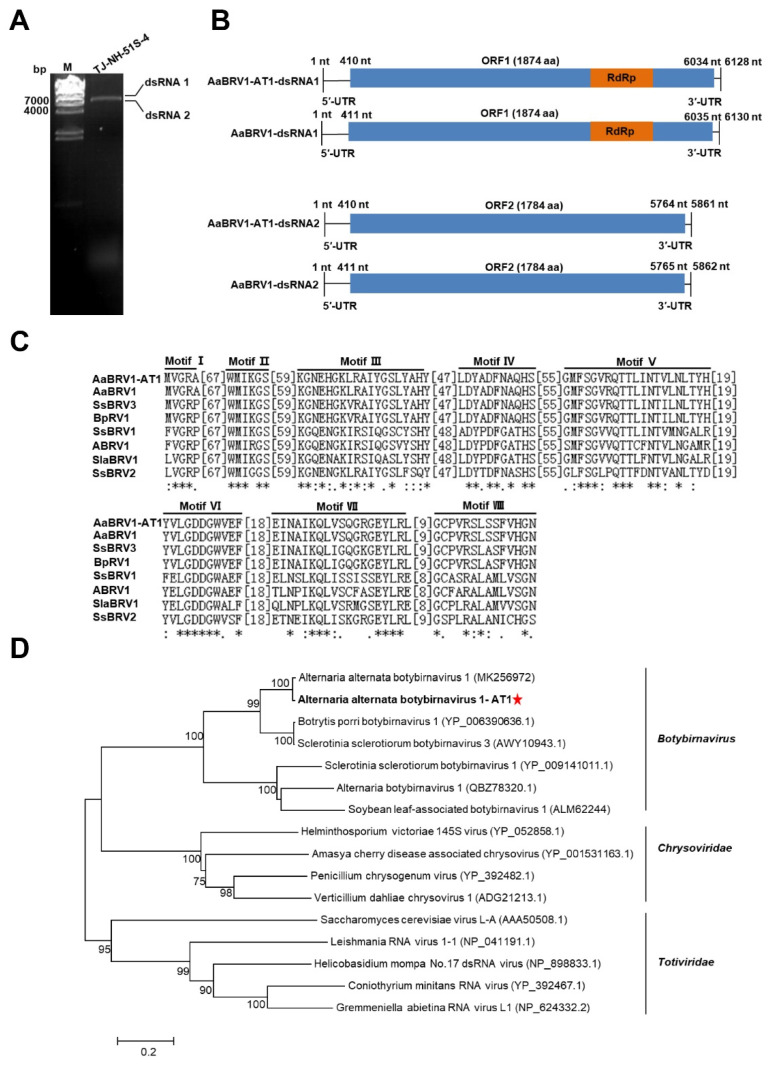
Characterization of the mycovirus Alternaria alternata botybirnavirus 1-AT1 (AaBRV1-AT1) in *Alternaria tenuissima* strain TJ-NH-51S-4. (**A**) Gel electrophoretic profiles of double-stranded RNA (dsRNA) extracted from strain TJ-NH-51S-4, which was treated with DNase I and S1 Nuclease (M: λ-*Hin*d III digest DNA marker). (**B**) Schematic diagram of the genomic organization of AaBRV1-AT1 and Alternaria alternata botybirnavirus 1 (AaBRV1). Open reading frames (ORFs) and untranslated regions (UTRs) are indicated by rectangles and single lines, respectively. The orange bar indicates conserved domains of RNA-dependent RNA polymerase (RdRp). (**C**) Multiple alignments of amino acid (aa) sequences of the RdRps in AaBRV1-AT1 and representative members of the genus *Botybirnavirus*. Eight conserved motifs (motif I to motif VIII) were identified in AaBRV1-AT1 and other members of the genus *Botybirnavirus*. Asterisks represent identical amino acid residues, colons represent amino acids with a high level of chemical similarity, and dots represent amino acid residues with a low of chemical similarity. (**D**) Phylogenetic tree based on the deduced aa sequences of the putative RdRps in AaBRV1-AT1 and other members of the genus *Botybirnavirus* using the maximum-likelihood (ML) method with 1000 bootstrap replicates. Bar scale represents a genetic distance of 0.2 aa substitutions per site. Red star indicates the position of AaBRV1-AT1.

The dsRNA extracted from *A. tenuissima* strain TJ-NH-51S-4 was electrophoresed in a 1.0% (*w*/*v*) agarose gel, and two bands (each approximately 6.0 kb) were observed, as would be expected for the genome of botybirnaviruses (Figure 1A). The complete nucleotide sequence of the two dsRNAs was determined to be 6128 bp (dsRNA1) with a G+C content of 49.33% and 5861 bp (dsRNA2) with a G+C content of 49.22%, respectively. The genome sequences were deposited in GenBank under the accession numbers OM371000 and OM371001. The ORF1 in dsRNA1 was expected to encode a polypeptide of 1874 aa residues containing a conserved domain of RdRp and was 93.65% identical to its counterpart in AaBRV1 (Figure 1B). The ORF2 in dsRNA2 encoded a 1784-aa polypeptide with 94.56% identity to the putative protein encoded by ORF2 in AaBRV1 (Figure 1B). RdRp in AaBRV1-AT1 also had eight conserved motifs (motif I to motif VIII) that are characteristic of RdRp in botybirnaviruses (Figure 1C). A phylogenetic tree based on the aa sequences of RdRp of representative members in the genera *Botybirnavirus*, *Chrysovirus*, and *Totivirus* revealed that AaBRV1-AT1 clustered together with members in the genus *Botybirnavirus* in the same clade and was the most closely related to AaBRV1 (Figure 1D). Collectively, these results indicate that AaBRV1-AT1 is a novel strain of AaBRV1.

### 3.2. Effect of AaBRV1-AT1 on the Phenotype of Its Host Fungus A. tenuissima

**Figure 2:**
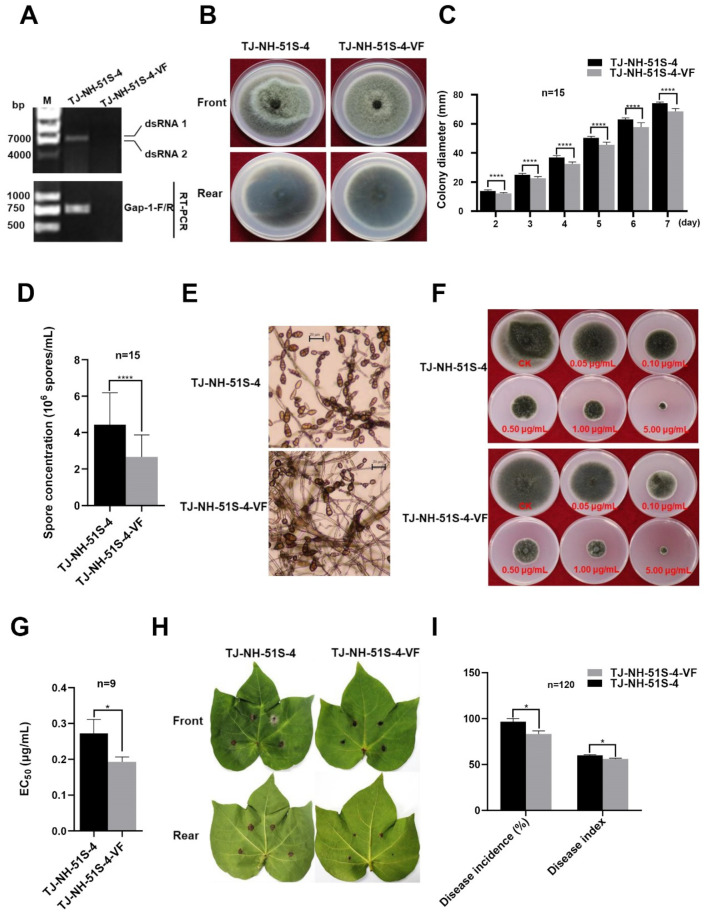
Colony morphology, colony growth rate, spore production, sensitivity to difenoconazole, and virulence of *Alternaria tenuissima* strains TJ-NH-51S-4 and TJ-NH-51S-4-VF. (**A**) Confirmation of the presence or absence of Alternaria alternata botybirnavirus 1-AT1 (AaBRV1-AT1) in strains TJ-NH-51S-4 and TJ-NH-51S-4-VF, respectively, by dsRNA profiles (top) from the two strains using agarose gel electrophoresis (M: λ-*Hin*d III digest DNA marker) and RT-PCR analysis using virus-specific primers (bottom) (M: DNA molecular marker DL 2000). (**B**) Colony morphology of strains TJ-NH-51S-4 and TJ-NH-51S-4-VF cultured on potato dextrose agar (PDA) plates at 25 °C for 7 d in darkness. (**C**) Colony diameter measured over 2−7 d of strains TJ-NH-51S-4 and TJ-NH-51S-4-VF cultured on PDA plates at 25 °C in darkness. (**D**) Conidiophores produced by strains TJ-NH-51S-4 and TJ-NH-51S-4-VF cultured on PDA plates at 25 °C for 7 d in darkness as viewed with a Nikon Eclipse Ci microscope. (**E**) Spore concentration in strains TJ-NH-51S-4 and TJ-NH-51S-4-VF cultured on PDA plates at 25 °C for 7 d in darkness. (**F**) Effect of difenoconazole on colony growth of strains TJ-NH-51S-4 and TJ-NH-51S-4-VF. (**G**) Median effective concentration (EC_50_) of difenoconazole against strains TJ-NH-51S-4 and TJ-NH-51S-4-VF. (**H**) Disease symptoms on cotton leaves inoculated with strains TJ-NH-51S-4 and TJ-NH-51S-4-VF at 7 d post inoculation. (**I**) Disease incidence and disease index on cotton leaves inoculated with strains TJ-NH-51S-4 and TJ-NH-51S-4-VF. Stars indicate different levels of significant difference between the two strains as determined by paired *t*-test using Graphpad Prism version 8.0 software (*, *p* < 0.05; ****, *p* < 0.0001).

The mycovirus AaBRV1-AT1-free strain TJ-NH-51S-4-VF was successfully obtained using protoplast regeneration to eliminate AaBRV1-AT1 from the mycovirus AaBRV1-AT1-infected strain TJ-NH-51S-4 (Figure 2A). TJ-NH-51S-4-VF was demonstrated to be AaBRV1-AT1-free by gel electrophoretic profiles of dsRNA and by RT-PCR analysis using AaBRV1-AT1-specific primers (Table 1).

The color of strain TJ-NH-51S-4 colonies was brown after being grown on PDA plates at 25 °C in the dark for 7 d, while the color of strain TJ-NH-51S-VF colonies was gray-green. The colony shape in strain TJ-NH-51S-4 was irregular in contrast to strain TJ-NH-51S-VF, which exhibited regular-shaped colonies (Figure 2B). The average colony growth rate of strain TJ-NH-51S-4 (10.60 mm/d) was significantly higher than it was in strain TJ-NH-51S-VF (9.79 mm/d) (Figure 2C). Average spore concentrations produced by strains TJ-NH-51S-4 and TJ-NH-51S-4-VF were 4.43 × 10^6^ spores/mL and 2.67 × 10^6^ spores/mL (Figure 2D), respectively, with the spore concentration of the former being significantly higher than it was in the latter (Figure 2E). Collectively, the effect of AaBRV1-AT1 infection on the phenotype of its host fungus *A. tenuissima* strain TJ-NH-51S-4 included altered colony morphology, increased colony growth, and an increased ability to produce spores (Figure 2B−E).

Difenoconazole inhibited colony growth of both TJ-NH-51S-4 and TJ-NH-51S-4-VF (Figure 2F); however, the EC_50_ value of difenoconazole against strain TJ-NH-51S-4 (0.2726 μg/mL) was significantly higher than it was against TJ-NH-51S-VF (0.1929 μg/mL) (Figure 2G). These data indicate that the sensitivity of strain TJ-NH-51S-4 to difenoconazole decreased following AaBRV1-AT1 infection.

Cotton leaves inoculated with strains TJ-NH-51S-4 and TJ-NH-51S-VF all exhibited dark brown circular lesions at 7 d post inoculation; however, the diameter of lesions on cotton leaves inoculated with strain TJ-NH-51S-4 was larger than the diameter of lesions on cotton leaves inoculated with strain TJ-NH-51S-VF (Figure 2H). Statistical analysis indicated that the disease incidence and the disease index (96.67% and 48.61, respectively) in cotton leaves inoculated with strain TJ-NH-51S-4 were much higher than those (83.33% and 44.45, respectively) in cotton leaves inoculated with strain TJ-NH-51S-4-VF (Figure 2I). Thus, we concluded that AaBRV1-AT1 infection of TJ-NH-51S-4 confers hypervirulence.

### 3.3. Vertical and Horizontal Transmission of AaBRV1-AT1

**Figure 3:**
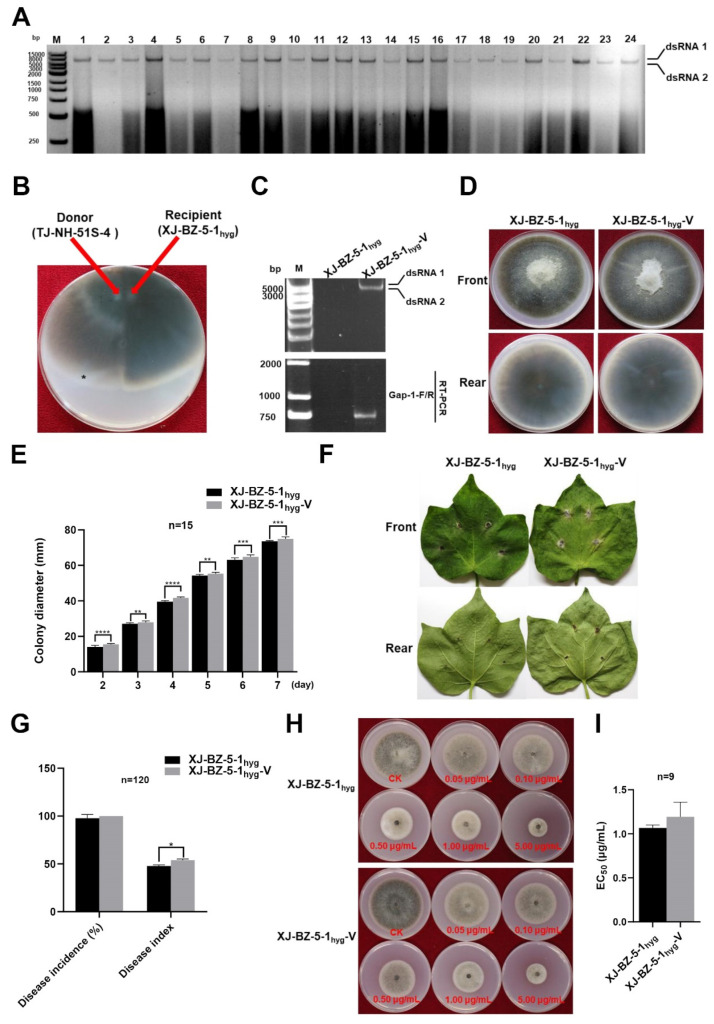
Vertical transmission via asexual spores and horizontal transmission through hyphal contact in pairing cultures of the mycovirus Alternaria alternata botybirnavirus 1-AT1 (AaBRV1-AT1). (**A**) Validation of positive presence of AaBRV1-AT1 in the 24 single-spore cultures derived from strain TJ-NH-51S-4 by dsRNA profiles using agarose gel electrophoresis (M: 250 bp DNA Ladder). (**B**) Pairing cultures of colonies of *Alternaria tenuissima* strain TJ-NH-51S-4 (donor strain, on the left) and *A. tenuissima* strain XJ-BZ-5-1_hyg_ (recipient strain, on the right) on the same PDA plate. The symbol ‘*’ over the colony indicates the location where a mycelial agar plug was collected and transferred to fresh PDA plates to obtain strain XJ-BZ-5-1_hyg_-V carrying AaBRV1-AT1 (AaBRV1-AT1 was horizontally transmitted from strain TJ-NH-51S-4 to strain XJ-BZ-5-1_hyg_ to obtain XJ-BZ-5-1_hyg_-V). (**C**) Confirmation of AaBRV1-AT1 in strains XJ-BZ-5-1_hyg_ and XJ-BZ-5-1_hyg_-V by dsRNA profiles (top) extracted from the two strains and separated using agarose gel electrophoresis (M: DNA molecular marker DL 5000) and RT-PCR analysis using mycovirus-specific primers (bottom) (M: DNA molecular marker DL 2000). (**D**) Colony morphology of strains XJ-BZ-5-1_hyg_-V and XJ-BZ-5-1_hyg_ cultured on PDA plates at 25 °C for 7 d in the dark. (**E**) Colony diameter of strains XJ-BZ-5-1_hyg_-V and XJ-BZ-5-1_hyg_. (**F**) Disease symptoms on cotton leaves inoculated with strains XJ-BZ-5-1_hyg_-V and XJ-BZ-5-1_hyg_. (**G**) Disease incidence and disease index on cotton leaves inoculated with strains XJ-BZ-5-1_hyg_-V and XJ-BZ-5-1_hyg_ at 7 d post inoculation. (**H**) Effect of difenoconazole on colony growth of strains XJ-BZ-5-1_hyg_-V and XJ-BZ-5-1_hyg_. (**I**) Median effective concentration (EC_50_) of difenoconazole against strains XJ-BZ-5-1_hyg_-V and XJ-BZ-5-1_hyg_. Stars indicate different levels of significant difference between the two strains as determined by paired *t*-test using Graphpad Prism version 8.0 software (*, *p* < 0.05; **, *p* < 0.01; ***, *p* < 0.001; ****, *p* < 0.0001).

Gel electrophoretic profiles of dsRNA extracted from 24 single-spore cultures derived from strain TJ-NH-51S-4 were evaluated. Results indicated 100% vertical transmission of AaBRV1-AT1 via asexual spores (Figure 3A).

Pairing cultures of donor strain TJ-NH-51S-4 and recipient strain XJ-BZ-5-1_hyg_ on PDA plates were used in the horizontal transmission assays (Figure 3B). As a result of the pairing, strain XJ-BZ-5-1_hyg_-V carrying the mycovirus AaBRV1-AT1 was obtained and verified (Figure 3C) by gel electrophoretic profiles of dsRNA and the detection of mycovirus AaBRV1-AT1 by RT-PCR using specific primers (Table 1). The colony morphology of the two strains (XJ-BZ-5-1_hyg_ and XJ-BZ-5-1_hyg_-V) were nearly identical (Figure 3D). The average colony growth rate (10.72 mm/d) of strain XJ-BZ-5-1_hyg_-V was significantly higher than that (10.50 mm/d) of strain XJ-BZ-5-1_hyg_ (Figure 3E). Cotton leaves inoculated with strains XJ-BZ-5-1_hyg_-V and XJ-BZ-5-1_hyg_ exhibited dark brown circular lesions that were similar in color and size (Figure 3F), and disease incidence was also nearly the same (Figure 3G). The disease index (53.90) on cotton leaves inoculated with strain XJ-BZ-5-1_hyg_-V was significantly higher than it (47.80) was on cotton leaves inoculated with strain XJ-BZ-5-1_hyg_ (Figure 3G). The EC_50_ of difenoconazole against strain XJ-BZ-5-1_hyg_-V was 1.1928μg/mL, while the EC_50_ of difenoconazole against strain XJ-BZ-5-1_hyg_ was 1.0671 μg/mL (Figure 3H−I). Thus, the data indicated that AaBRV1-AT1 could be transmitted horizontally and that the effect of AaBRV1-AT1 infection on the phenotype of strain TJ-NH-51S-4 and XJ-BZ-5-1_hyg_-V were similar.

### 3.4. Differentially Expressed Genes (DEGs)

**Figure 4:**
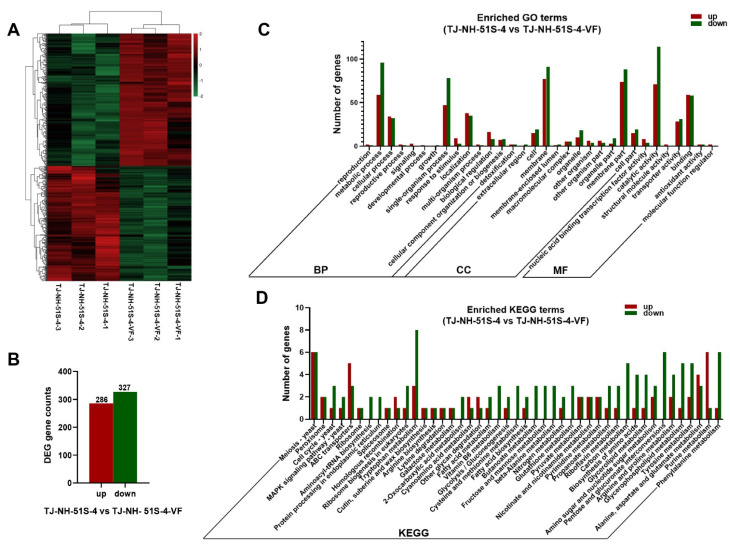
Transcriptional analyses of differentially expressed genes (DEGs) in *Alternaria tenuissima* strains TJ-NH-51S-4 and TJ-NH-51S-4-VF. (**A**) Hierarchical clustering of DEGs in strains TJ-NH-51S-4 and TJ-NH-51S-4-VF. Red and green colors indicate varying degrees of up-regulated and down-regulated genes, respectively. Expression key is indicated on the upper right side of the figure. (**B**) A total of 613 DEGs were identified, of which 286 DEGs were up-regulated, and 327 DEGs were down-regulated. (**C**) Gene Ontology (GO) classification analysis of DEGs in strains TJ-NH-51S-4 and TJ-NH-51S-4-VF. (**D**) Kyoto Encyclopedia of Genes and Genomes (KEGG) pathways classification analysis of DEGs in strains TJ-NH-51S-4 and TJ-NH-51S-4-VF.

Genome-wide differences in transcription between strains TJ-NH-51S-4 and TJ-NH-51S-4-VF were investigated. A heat map depicting the relative expression level of DEGs between strains TJ-NH-51S-4 (three biological replicates, namely TJ-NH-51S-4-1, TJ-NH-51S-4-2, and TJ-NH-51S-4-3) and TJ-NH-51S-4-VF (three biological replicates, namely TJ-NH-51S-4-VF-1, TJ-NH-51S-4-VF-2, and TJ-NH-51S-4-VF-3) is presented in Figure 4A. The analysis of the transcriptome data comparing gene expression in TJ-NH-51S-4 vs. TJ-NH-51S-4-VF identified a total of 613 DEGs, among which 286 (46.66%) were up-regulated and 327 (53.34%) were down-regulated in strain TJ-NH-51S-4 (Figure 4B).

Three hundred and seventy-four of the DEGs were annotated in the GO analysis (59.27%) and classified into three major functional ontologies, namely biological process, cellular component, and molecular function (Figure 4C). Based on low to high *p* values, the GO term “metabolic process” (GO: 0008152) had the highest number (155 genes) of significantly enriched genes in biological process, while the GO term “catalytic activity” (GO: 0003824) had the highest number (185 genes) in molecular function, and the GO term “membrane” (GO: 0016020) had the highest number (162 genes) in cellular component (Table S2). A total of 192 DEGs were annotated to KEGG pathways. The top three enriched terms were “Meiosis-yeast” (ko04113), “Tryptophan metabolism” (ko00380), and “ABC transporters” (ko02010) (Figure 4D and Table S3). Among 10 DEGs associated with amino acid metabolism (including alanine, aspartic acid, glutamic acid, tyrosine, and tryptophan metabolism), 6 DEGs were up-regulated 2.2-fold to 4.9-fold, and 4 DEGs were down-regulated 2.2-fold to 5.1-fold (Table S4).

Among 12 DEGs related to the cytochrome P450 gene family, 3 DEGs were up-regulated 2.5-fold to 3.3-fold, and 9 DEGs were down-regulated 2.1-fold to 4.4- fold (Table S5). Notably, gene At-g11265 encoding CYP51 was up-regulated 2.9-fold in strain TJ-NH-51S-4 relative to strain TJ-NH-51S-4-VF.

DEGs identified to encode an azole resistance protein, an aminotriazole resistance protein, and a multidrug resistance protein were significantly down-regulated 21.2-fold, 7.6-fold, and 3.5-fold, respectively (Table S5). Only one of the down-regulated DEGs was assigned to ABC transporters according to the NR database (Table S5), and six DEGs were annotated to MFS transporters, one of which was up-regulated 2.4-fold and five of which were down-regulated 2.8-fold to 6.5-fold. Among three DEGs related to other putative transporters, one was up-regulated 3.0-fold, and the other two were down-regulated 2.2-fold and 7.8-fold, respectively. Additionally, one DEG related to a membrane transporter was down-regulated 10.2-fold (Table S5), and DEGs assigned to siderophore iron transporters (SIT) belonging to major facilitator systems (MFS) were up-regulated 3.1-fold to 4.5-fold (Table S5).

**Figure 5:**
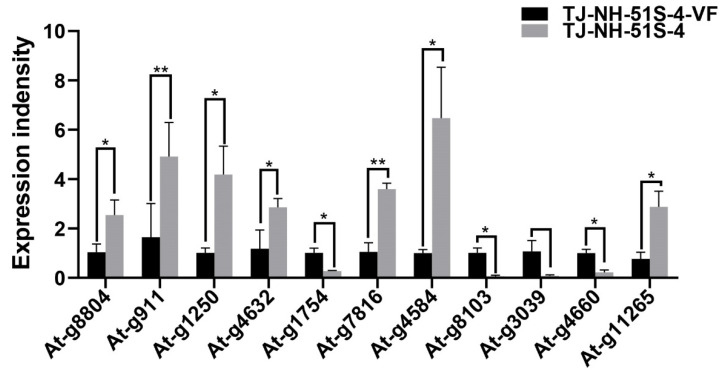
Validation of the RNA sequence (RNA-seq) data obtained for *Alternaria tenuissima* strains TJ-NH-51S-4 and TJ-NH-51S-4-VF using reverse transcription-quantitative polymerase chain reaction (RT-qPCR). Information on the eleven differentially expressed genes (DEGs) is provided in detail in Table S5. Stars indicate different levels of significant difference between the two strains as determined by paired *t*-test using Graphpad Prism version 8.0 software (*, *p* < 0.05; **, *p* < 0.01).

Ten DEGs chosen at random representing different intracellular functions and the gene At-g11265 (Table S5) were subjected to RT-qPCR analysis to validate the expression results obtained in the RNA-seq data. Results of the RT-qPCR analysis indicated that among the 10 DEGs, the expression of 6 DEGs was up-regulated 2.5-fold to 10.4-fold, and 4 were down-regulated 10.2-fold to 3.8-fold in TJ-NH-51S-4, relative to their expression in TJ-NH-51S-4-VF (Figure 5 and Table S5), which was consistent with the RNA-seq data. Results of the RT-qPCR analysis also indicated that the expression level of At-g11265 encoding CYP51 in strain TJ-NH-51S-4 was up-regulated relative to strain TJ-NH-51S-4-VF (Figure 5 and Table S5), which was also in accordance with the results obtained in the RNA-seq data.

## 4. Discussion

In the present study, a botybirnavirus was isolated from *A. tenuissima* strain TJ-NH-51S-4, the causal agent of cotton Alternaria leaf spot, whose genomic organization is the most similar to AaBRV1 isolated from *A. alternata*, the causal agent of watermelon leaf blight [10]. The aa sequences of proteins encoded by ORF1 in dsRNA1 and ORF2 in dsRNA2 of the mycovirus had the highest identity (93.65% and 94.56%, respectively) with the corresponding sequences in AaBRV1. Moreover, the mycovirus isolated from strain TJ-NH-51S-4 in this study and AaBRV1 clustered closely together in the same clade in a phylogenetic tree with strong bootstrap support. Collectively, the mycovirus appeared to be a new strain of AaBRV1 and designated as AaBRV1-AT1. Botybirnaviruses have been previously identified from a phytopathogenic *Alternaria* fungus infecting pear [9] and *A. alternata* infecting watermelon [10]; however, to our knowledge, this is the first report of a botybirnavirus that infects *A. tenuissima*.

The majority of mycoviruses do not have a visible effect on their fungal hosts [11,12]. Alterations in fungal morphology, however, may be an indicator of mycovirus infection. For example, CHV-1-infected isolates of *C. parasitica* lack the characteristic orange pigment [13], *S. sclerotiorum* isolates infected by SsHADV-1 exhibit abnormal morphology and produce only a few small sclerotia [14], and Alternaria alternata chrysovirus 1 (AaCV-1) infection alters the morphology and pigment production of *A. alternata* [19]. Additionally, some fungi, including *R. solani*, *Aspergillus fumigatus*, *A. alternata*, and *T. marneffe*, exhibit mycovirus-mediated hypervirulence [16,17,18,19,20]. The effect of mycovirus on host virulence has been reported to be potentially accompanied by alterations in the sporulation and radial growth rate of the host fungus [54]. Infection of isolates of *Nectria radicicola* with a 6 kb dsRNA conferred hypervirulence and enhanced sporulation [55]. Beauveria bassiana polymycovirus-1 (BbPmV-1) and Beauveria bassiana non-segmented virus-1(BbNV-1) were reported to not only confer hypervirulence to their hosts but also increase the colony growth rate of their host fungi [56]. Notably, five mycoviruses in the genus *Botybirnavirus*, namely BpRV1, SsBRV2, BmBRV1-BdEW220, SsBRV1, and ABRV1, have been previously reported to confer hypovirulence to their hosts [3,4,5,7,9]. In the present study, significant changes in the host fungus, *A. tenuissima*, were found following AaBRV1-AT1 infection, including abnormal morphology, enhanced colony growth and spore production, and hypervirulence. Notably, this is the first report of hypervirulence in the phytopathogenic fungus *A. tenuissima*, and perhaps the entire genus of *Alternaria*, caused by infection of a botybirnavirus.

Co-infection of Penicillium digitatum polymycovirus 1 (PdPmV1) and Penicillium digitatum Narna-like virus 1 (PdNLV1) [29] and a single infection of Penicillium crustosum chrysovirus 1 (PcCV1) [30] were both shown to enhance the sensitivity of their host fungi (*Penicillium digitatum* and *P. crustosum*) to prochloraz. In contrast, AaCV1-AT1 infection of *A. tenuissima* reduced host sensitivity to difenoconazole and tebuconazole [31]. In the present study, AaBRV1-AT1 infection decreased the sensitivity of its host fungus *A. tenuissima* strain TJ-NH-51S-4 to difenoconazole, similar to the situation in AaCV1-AT1 infection of *A. tenuissima*.

Transmission of mycoviruses principally occurs through sporogenesis and hyphal anastomosis, with hyphal anastomosis naturally occurring between individuals of closely related vegetative compatibility groups [57]. BmBRV1-BdEW220, a member of the genus *Botybirnavirus*, was shown to be transmitted vertically via asexual spores [7]. Sclerotinia sclerotiorum partitivirus 1 (SsPV1/WF-1) was readily transmitted horizontally through hyphal contact with different vegetative compatibility groups in *S. sclerotiorum* [57]. In the current study, AaBRV1-AT1 was also transmitted vertically via asexual spores and transmitted horizontally from the AaBRV1-AT1-infected strain TJ-NH-51S-4 to the AaBRV1-AT1-free strain XJ-BZ-5-1_hyg_ (another strain of *A. tenuissima*) through hyphal contact in pairing cultures. AaBRV1 was isolated from *A. alternata* in our previous study [10], as well as from *A. tenuissima* in this study, representing two taxonomically distinct fungi in the genus *Alternaria*. Thus, we hypothesize that AaBRV1 may be able to overcome vegetative incompatibility in different species of *Alternaria.* Further studies are needed, however, to determine if AaBRV1-AT1 can be transmitted horizontally from *A. tenuissima* to another species of *Alternaria*, such as *A. alternata*.

SIT is a unique fungal protein and part of MFS, which functions as a proton co-transporter across membranes [58,59] and is involved in the regulation of pathogenicity and drug resistance in pathogenic fungi [60]. All eukaryotes and most prokaryotes require iron as a micronutrient, and the uptake of iron is critical for opportunistic fungi and represents a crucial pathogenic factor [61]. In the present study, four DEGs related to SIT were found to be up-regulated in *A. tenuissima* strain TJ-NH-51S-4 infected with AaBRV1-AT1 and, thus, may potentially increase the virulence of this pathogen.

Cytochrome P450 family genes catalyze the production of several fungal secondary metabolites and contribute to the biosynthesis of a variety of mycotoxins typically linked to pathogenic processes, including drug resistance and cell growth, defense, and detoxification [62], which are activated during the development of plant diseases [63,64]. Cytochrome P450 is also involved in metabolizing pesticides, predominantly mediated by monooxygenase [65]. In the present study, the differential expression of genes related to cytochrome P450 in AaBRV1-AT1-infected strain TJ-NH-51S-4 appears to affect both the primary and secondary metabolism of *A. tenuissima*.

Previous studies have indicated that overexpression of CYP51, ABC transporter, and/or MFS in fungi enhances drug resistance, including DMI-resistance, and thus reduces fungal sensitivity to fungicides, including DMIs [26,27,28]. In our study, one DEG-encoding CYP51 was significantly up-regulated in TJ-NH-51S-4, while one DEG related to ABC transporter and six DEGs related to MFS transporters were all down-regulated. Therefore, we hypothesize that the reduced sensitivity of *A. tenuissima* infected with AaBRV1-AT1 to difenoconazole may be due to the up-regulation of CYP51 target-enzyme genes.

Our findings in the present study provide a better understanding of the effect of the mycovirus AaBRV1-AT1 infection on the biological properties of its host fungus *A. tenuissima* strain TJ-NH-51S-4, as well as the potential mechanisms responsible for hypervirulence and a decreased sensitivity to difenoconazole. Our study also identified a valuable experimental system that can be used to study the interaction between botybirnaviruses and their fungal hosts. The utilization of gene knockdown and genetic transformation technologies will be used in future studies to confirm the functional impact of the identified DEGs on the biological properties of *A. tenuissima* resulting from its infection by the mycovirus, AaBRV1-AT1.

## Figures and Tables

**Table 1:** Primers used to determine the complete genome sequence of the mycovirus Alternaria alternata botybirnavirus 1-AT1 (AaBRV1-AT1) in this study.

Primer Name	Sequence (5′-3′)
RACE3	CGATCGATCATGATGCAATGC
RACE3RT	CGATCGATCATGATGCAATGCNNNNNN
PC3-T7 Loop adapter	p-GGATCCCGGGAATTCGGTAATACGACTCA CTATATTTTTATAGTGAGTCGTATTA-OH
PC2	CCGAATTCCCGGGATCC
AaBRV1-AT1-dsRNA1-3end	TAACAAGTTCAAAGCATCTGGAG
AaBRV1-AT1-dsRNA1-5end	TGGGAGATTACAGGTGGCTTCA
AaBRV1-AT1-dsRNA2-3end	CAGATTCAATGCCCACTGTAAG
AaBRV1-AT1-dsRNA2-5end	AGATGTTGGGAGATTACAGGTGG
AaBRV1-AT1-dsRNA1-Gap -1-F	AATCGTATGGAAGGGTAA
AaBRV1-AT1-dsRNA1-Gap -1-R	TACTTGAAGTCGGTGGTG
AaBRV1-AT1-dsRNA2-Gap -2-F	TGCGTAGTCCAGATTGCCG

## Data Availability

The sequences reported in the present manuscript have been deposited in the GenBank database under accession numbers OM371000 and OM371001. The RNA-seq raw data from the three biological replicates of strains TJ-NH-51S-4 and TJ-NH-51S-4-VF were deposited in the NCBI Sequence Read Archive (SRA) database under the accession number PRJNA880535.

## Supplementary Materials

The following are available online at https://www.mdpi.com/article/10.3390/v14102093/s1. Table S1. Primers used for reverse transcription-quantitative polymerase chain reaction (RT-qPCR) analysis of differentially expressed genes (DEGs) identified in the RNA sequencing (RNA-seq) data. Table S2. Gene Ontology (GO) annotation of differentially expressed genes (DEGs) identified in the comparison between *Alternaria tenuissima* strains TJ-NH-51S-4 and TJ-NH-51S-4-VF (False discovery rate <0.05). Table S3. Kyoto Encyclopedia of Genes and Genomes (KEGG) annotation of differentially expressed genes (DEGs) identified in the comparison between *Alternaria tenuissima* strains TJ-NH-51S-4 and TJ-NH-51S-4-VF. Table S4. The Log_2_fold change in differentially expressed genes (DEGs) related to amino acid metabolism in *Alternaria tenuissima* strain TJ-NH-51S-4, relative to *A. tenuissima* strain TJ-NH-51S-4-VF, and their annotation (RefSeq non-redundant proteins (NR) and protein family database (Pfam)). Table S5. Validation of the expressed change ratio Log_2_FoldChange, relative to *Alternaria tenuissima* strain TJ-NH-51S-4-VF obtained in the RNA sequencing (RNA-seq) data using reverse transcription-quantitative polymerase chain reaction (RT-qPCR). Listed genes are differentially expressed genes (DEGs) related to cytochrome P450, drug resistance, ATP-binding cassette transporter (ABC), and major facilitator superfamily (MFS) in *A. tenuissima* strain TJ-NH-51S-4 as annotated in RefSeq non-redundant proteins (NR) and protein family (Pfam) databases. Eleven DEGs were selected for the RT-qPCR validation of the RNA-seq data.
