# Supplementary material for: A Botybirnavirus Isolated from Alternaria tenuissima Confers Hypervirulence and Decreased Sensitivity of Its Host Fungus to Difenoconazole
Source: Viruses. 2022 Sep 21;14(10):2093. doi: 10.3390/v14102093 (PMC9611228; doi:10.3390/v14102093)
Supplement: Supplementary file 1 [file viruses-14-02093-s001.zip › Table S1.pdf]

Table S1. Primers used for reverse transcription-quantitative polymerase chain reaction (RT-qPCR) analysis of differentially expressed genes (DEGs) identified in the RNA sequencing (RNA-seq) data.

Gene name	Primer name	Sequence (5′-3′)
At-g8804	g8804-1F	ATCTTCAATGCGGATGGCG
	g8804-1R	GGAGCGAGTTGGTAGAGTGGTT
At-g911	g911-1F	CTGGGCGAATCAAAGACTGC
	g911-1R	TTCCGAACTTCCAATGGTAAA
At-g1250	g1250-1F	CATTTTGACGGTGAGTTGGC
	g1250-1R	CAGCAGATAGAAGGGAAGCATT
At-g4632	g4632-1F	CGCTTTTACCTCTTCGCCATA
	g4632-1R	CCAGTCTGCTTCGCCTTCC
At-g1754	g1754-1F	CACAATACTGTCCAATCAGGGTC
	g1754-1R	GTGTAGGAGTGCGATGAGAAGA
At-g7816	g7816-1F	AAATGCTCGCCTTCACCTG
	g7816-1R	AGACCCTTGCCCTTGTTCC
At-g4584	g4584-1F	ATTGAAGAGGAGGGTATTGCG
	g4584-1R	CGTCACCAGGGTTCCAGTTAG
At-g8103	g8103-1F	AGATGACCCCGAAAACCCC
	g8103-1R	AGATGACCCCGAAAACCCC
At-g3039	g3039-1F	AACATAACGAGCGGCAATAAC
	g3039-1R	TTGAGTGCTGGAAGGAACATAG
At-g4660	g4660-1F	CAGGGCGTTCTTAGCGATGT
	g4660-1R	GGTGCGTATGTTTCTTGGAGG
At-g11265	g11265-1F	CGCTCAGGTCGTCAAGGAA
	g11265-1R	ACTGTTGGTGCTCGGGGTC
Histone 3	H3-1a	ACTAAGCAGACCGCCCGCAGG
	H3-1b	GCGGGCGAGCTGGATGTCCTT
