# Supplementary material for: A Botybirnavirus Isolated from Alternaria tenuissima Confers Hypervirulence and Decreased Sensitivity of Its Host Fungus to Difenoconazole
Source: Viruses. 2022 Sep 21;14(10):2093. doi: 10.3390/v14102093 (PMC9611228; doi:10.3390/v14102093)
Supplement: Supplementary file 1 [file viruses-14-02093-s001.zip › Table S2.pdf]

Table S2. Gene Ontology (GO) annotation of differentially expressed genes (DEGs) identified in the comparison between *Alternaria tenuissima* strains TJ-NH-51S-4 and TJ-NH-51S-4-VF (False discovery rate <0.05).

Term_type	GO_accession	Description	Number of Up-regulated genes	Number of Down-regulated genes
Biological process	GO:0000003	Reproduction	2	1
Biological process	GO:0008152	Metabolic process	59	96
Biological process	GO:0009987	Cellular process	34	32
Biological process	GO:0022414	Reproductive process	2	1
Biological process	GO:0023052	Signaling	3	0
Biological process	GO:0032502	Developmental process	1	0
Biological process	GO:0040007	Growth	0	1
Biological process	GO:0044699	Single-organism process	47	78
Biological process	GO:0050896	Response to stimulus	9	3
Biological process	GO:0051179	Localization	38	35
Biological process	GO:0051704	Multi-organism process	2	1
Biological process	GO:0065007	Biological regulation	16	8
Biological process	GO:0071840	Cellular component organization or biogenesis	7	8
Biological process	GO:0098754	Detoxification	2	2

Cellular component	GO:0005576	Extracellular region	0	2
Cellular component	GO:0005623	Cell	15	19
Cellular component	GO:0016020	Membrane	77	91
Cellular component	GO:0031974	Membrane-enclosed lumen	1	2
Cellular component	GO:0032991	Macromolecular complex	5	5
Cellular component	GO:0043226	Organelle	10	18
Cellular component	GO:0044215	Other organism	6	4
Cellular component	GO:0044217	Other organism part	6	4
Cellular component	GO:0044422	Organelle part	3	9
Cellular component	GO:0044425	Membrane part	74	88
Cellular component	GO:0044464	Cell part	15	19
Cellular component	GO:0003700	Nucleic acid binding transcription factor activity	8	4
Cellular component	GO:0003824	Catalytic activity	71	114
Molecular function	GO:0005198	Structural molecule activity	2	0
Molecular function	GO:0005215	Transporter activity	28	31
Molecular function	GO:0060090	Binding	59	58
Molecular function	GO:0016209	Antioxidant activity	2	2

Molecular function	GO:0098772	Molecular function regulator	2	0
