# Supplementary material for: A Botybirnavirus Isolated from Alternaria tenuissima Confers Hypervirulence and Decreased Sensitivity of Its Host Fungus to Difenoconazole
Source: Viruses. 2022 Sep 21;14(10):2093. doi: 10.3390/v14102093 (PMC9611228; doi:10.3390/v14102093)
Supplement: Supplementary file 1 [file viruses-14-02093-s001.zip › Table S3.pdf]

Table S3. Kyoto Encyclopedia of Genes and Genomes (KEGG) annotation of differentially expressed genes (DEGs) identified in the comparison between *Alternaria tenuissima* strains TJ-NH-51S-4 and TJ-NH-51S-4-VF.

**GO_accession**	**Description**	**Number of Up-regulated genes**	**Number of Down-regulated genes**
ko04113	Meiosis-yeast	6	6
ko04146	Peroxisome	2	2
ko04111	Cell cycle-yeast	1	3
ko04011	MAPK signaling pathway-yeast	1	2
ko02010	ABC transporters	5	3
ko03010	Ribosome	1	1
ko00970	Aminoacyl-tRNA biosynthesis	0	2
ko04141	Protein processing in endoplasmic reticulum	0	2
ko03040	Spliceosome	1	1
ko03440	Homologous recombination	2	1
ko03008	Ribosome biogenesis in eukaryotes	1	3
ko00380	Tryptophan metabolism	3	8
ko00073	Cutin, suberine, and wax biosynthesis	1	1
ko00220	Arginine biosynthesis	1	1
ko00310	Lysine degradation	1	1
ko00052	Galactose metabolism	1	1
ko01210	2-Oxocarboxylic acid metabolism	0	2
ko00460	Cyanoamino acid metabolism	2	1
ko00511	Other glycan degradation	2	1
ko01212	Fatty acid metabolism	1	2
ko00750	Vitamin B6 metabolism	0	3
ko00010	Glycolysis/Gluconeogenesis	1	2
ko00270	Cysteine and methionine metabolism	0	3

ko00061	Fatty acid biosynthesis	1	2
ko00650	Butanoate metabolism	0	3
ko00051	Fructose and mannose metabolism	0	3
ko00410	Beta-Alanine metabolism	0	3
ko00910	Nitrogen metabolism	1	2
ko00480	Glutathione metabolism	0	3
ko00620	Pyruvate metabolism	2	2
ko00760	Nicotinate and nicotinamide metabolism	2	2
ko00240	Pyrimidine metabolism	2	2
ko00640	Propanoate metabolism	1	3
ko00740	Riboflavin metabolism	1	3
ko01200	Carbon metabolism	0	5
ko01230	Biosynthesis of amino acids	1	4
ko00561	Glycerolipid metabolism	1	4
ko00520	Amino sugar and nucleotide sugar metabolism	2	3
ko00040	Pentose and glucuronate interconversions	0	6
ko00330	Arginine and proline metabolism	2	4
ko00564	Glycerophospholipid metabolism	1	5
ko00350	Tyrosine metabolism	2	5
ko00250	Alanine, aspartate, and glutamate metabolism	4	3
ko00230	Purine metabolism	6	1
ko00360	Phenylalanine metabolism	1	6
