# Supplementary material for: A Botybirnavirus Isolated from Alternaria tenuissima Confers Hypervirulence and Decreased Sensitivity of Its Host Fungus to Difenoconazole
Source: Viruses. 2022 Sep 21;14(10):2093. doi: 10.3390/v14102093 (PMC9611228; doi:10.3390/v14102093)
Supplement: Supplementary file 1 [file viruses-14-02093-s001.zip › Table S4.pdf]

Table S4. The Log$_2$fold change in differentially expressed genes (DEGs) related to amino acid metabolism in *Alternaria tenuissima* strain TJ-NH-51S-4, relative to *A. tenuissima* strain TJ-NH-51S-4-VF, and their annotation [RefSeq non-redundant proteins (NR) and protein family database (Pfam)].

**Gene_ID**	**NR description**	**Pfam description**	**Regulation model**	**Log$_2$Fold Change**
At-g2462	Acyl-CoA N-acyltransferase	Acetyltransferase (GNAT) family	up	2.2967
At-g3645	Di-copper center-containing protein	Common central domain of tyrosinase	up	1.8394
At-g5228	Glutamate dehydrogenase	Glutamate/Leucine/Phenylalanine/Valine dehydrogenase	up	1.6350
At-g8060	Glutamine--fructose-6-phosphate aminotransferase [isomerizing]	SIS domain	up	1.3150
At-g1037	Glutaryl-CoA dehydrogenase	Acyl-CoA dehydrogenase, C-terminal domain	up	1.2688
At-g6163	L-asparaginase 2-4	Asparaginase, N-terminal	up	1.1375
At-g9680	Glutamate decarboxylase	Pyridoxal-dependent decarboxylase conserved domain	down	-2.3412
At-g765	Phosphoesterase-like protein	Phosphoesterase family	down	-2.1685
At-g1621	Glutamate decarboxylase	Pyridoxal-dependent decarboxylase conserved domain	down	-1.7040
At-g4968	Alcohol dehydrogenase 2	Alcohol dehydrogenase GroES-like domain	down	-1.1130
