# Supplementary material for: A Botybirnavirus Isolated from Alternaria tenuissima Confers Hypervirulence and Decreased Sensitivity of Its Host Fungus to Difenoconazole
Source: Viruses. 2022 Sep 21;14(10):2093. doi: 10.3390/v14102093 (PMC9611228; doi:10.3390/v14102093)
Supplement: Supplementary file 1 [file viruses-14-02093-s001.zip › Table S5.pdf]

Table S5. Validation of the expressed change ratio [Log_2FoldChange, relative to *Alternaria tenuissima* strain TJ-NH-51S-4-VF] obtained in the RNA sequencing (RNA-seq) data using reverse transcription-quantitative polymerase chain reaction (RT-qPCR). Listed genes are differentially expressed genes (DEGs) related to cytochrome P450, drug resistance, ATP-binding cassette transporter (ABC), and major facilitator superfamily (MFS) in *A. tenuissima* strain TJ-NH-51S-4 as annotated in RefSeq non-redundant proteins (NR) and protein family (Pfam) databases. Eleven DEGs were selected for the RT-qPCR validation of the RNA-seq data.

**Gene_ID**	**NR description**	**Pfam description**	**Regulation model**	**RNA-seq (Log_2Fold Change)** [a]	**qRT-PCR ($2^{-\Delta\Delta ct}$)**	
					**TJ-NH-51S-4-VF**	**TJ-NH-51S-4**
At-g911	Cytochrome	Cytochrome P450	up	1.7289	1.6420	4.9130
At-g11265	Cytochrome P450 sterol 14α-demethylase (CYP51)	Cytochrome P450	up	1.5392	1.0913	2.7809
At-g8804	Cytochrome P450 family protein	Cytochrome P450	up	1.2978	1.0390	2.5480
At-g7231	Cytochrome P450 family protein	Cytochrome P450	down	-2.1232	— [b]	—
At-g1833	Cytochrome P450 family protein	Cytochrome P450	down	-1.9325	—	—
At-g1560	Cytochrome P450 52A12	Cytochrome P450	down	-1.8548	—	—
At-g5685	Cytochrome P450 family protein	Cytochrome P450	down	-1.6715	—	—
At-g1880	Cytochrome P450 family protein	Cytochrome P450	down	-1.6154	—	—

At-g10737	Cytochrome P450 family protein	Cytochrome P450	down	-1.5629	—	—
At-g610	Cytochrome P450 phenylacetate hydroxylase-like protein	Cytochrome P450	down	-1.3853	—	—
At-g3369	Cytochrome P450 3A17	Cytochrome P450	down	-1.3084	—	—
At-g3404	Cytochrome P450 76C3	Cytochrome P450	down	-1.0458	—	—
At-g9548	Azole resistance protein	Major facilitator superfamily	down	-4.4061	—	—
At-g9347	Aminotriazole resistance protein	Major facilitator superfamily	down	-2.9289	—	—
At-g5065	Multidrug resistance protein	ABC transporter transmembrane region	down	-1.7886	—	—
At-g1754	ATP-binding cassette transporter	ABC-2 type transporter	down	-1.9174	1.0125	0.2708
At-g6376	Putative transporter	Major facilitator superfamily	up	1.5736	—	—
At-g2640	MFS general substrate transporter	Major facilitator superfamily	up	1.2894	—	—
At-g8103	Membrane transporter	Major facilitator superfamily	down	-3.3514	1.0144	0.0645
At-g11728	Putative transporter	Major facilitator superfamily	down	-2.9717	—	—
At-g3039	MFS general substrate transporter	Major facilitator superfamily	down	-2.6997	1.0726	0.0753
At-g4660	MFS general substrate transporter	Major facilitator superfamily	down	-2.6934	1.0074	0.2227
At-g9794	MFS general substrate transporter	Major facilitator superfamily	down	-1.9477	—	—

At-g10416	MFS general substrate transporter	Major facilitator superfamily	down	-1.8821	—	—
At-g5043	MFS general substrate transporter	Major facilitator superfamily	down	-1.4775	—	—
At-g2973	Putative transporter	Major facilitator superfamily	down	-1.1134	—	—
At-g2452	Siderophore iron transporter	--	up	3.4328	—	—
At-g1250	Siderophore iron transporter	Major facilitator superfamily	up	2.1654	1.0136	4.1880
At-g7202	Siderophore iron transporter	Major facilitator superfamily	up	2.0584	—	—
At-g4632	Siderophore iron transporter 1	Major facilitator superfamily	up	1.6176	1.1762	2.8569
At-g7816	Phosphate permease	Phosphate transporter family	up	3.3835	1.0514	3.5930
At-g4584	Thioredoxin-like protein	AhpC/TSA family	up	2.0237	1.0064	6.4723

[a] The fold change values were originated from the data of RNA-seq which were evaluated using false discovery rate $P < 0.05$ and from the data of RT- qPCR.

[b] The symbol '—' showed that the DEGs were not chosen for validation of the data of RNA-seq.
]
